# Mental health challenges and perceived risks among female sex Workers in Nairobi, Kenya

**DOI:** 10.1186/s12889-022-14527-5

**Published:** 2022-11-24

**Authors:** Mamtuti Panneh, Mitzy Gafos, Emily Nyariki, Jennifer Liku, Pooja Shah, Rhoda Wanjiru, Mary Wanjiru, Alicja Beksinska, James Pollock, The Maisha Fiti Study Champions, Demtilla Gwala, Daisy Oside, Ruth Kamene, Agnes Watata, Agnes Atieno, Faith Njau, Elizabeth Njeri, Evelyn Orobi, Ibrahim Lwingi, Zaina Jama, Hellen Babu, Rupert Kaul, Janet Seeley, John Bradley, Joshua Kimani, Tara Beattie

**Affiliations:** 1 LSHTM, Department for Global Health and Development, London, UK; 2 Partners for Health and Development in Africa, Nairobi, Kenya; 3 Department of Immunology, University of Toronto, Toronto, Canada; 4 Department of Medicine, University of Toronto, Toronto, Canada; 5 MRC International Statistics and Epidemiology Group, Department for Infectious Disease Epidemiology, LSHTM, London, UK

**Keywords:** Mental health, Suicide, Intimate partner violence, Poverty, Female sex workers, Sex-work related risks

## Abstract

**Background:**

Female sex workers (FSWs) in Kenya are at an increased risk of violence, poverty, police arrest, and problematic alcohol and other substance use, all of which are linked to poor mental health and suicidal ideation. Despite the psychological stressors experienced by FSWs, there is no published qualitative methods research investigating their mental health experiences in Kenya. In this paper, we draw on data from in-depth interviews to examine FSWs’ lifetime mental health experiences and perceived risk factors.

**Methods:**

We used baseline in-depth interviews of the Maisha Fiti longitudinal study of FSWs in Nairobi. We randomly selected 40 FSWs from 1003 FSWs who attended a baseline behavioural-biological interview as part of the Maisha Fiti study. The interview guide was semi-structured, and participants were asked to detail their life stories, including narrating specific events such as entry into sex work, experiences of violence, mental health experiences, and use of alcohol and other substances. Interviews were recorded in Kiswahili/ English and transcribed in English. Data were coded and thematically analysed in Nvivo (v.12).

**Results:**

Results indicated that the majority of participants understood ‘mental health’ as ‘insanity’, ‘stress’, ‘depression’, and ‘suicide’; nevertheless, a number described mental health symptomatically, while a few believed that mental health problems were caused by witchcraft. Interestingly, poverty, low levels of education, poor job opportunities, a lack of family support, harmful gender norms, intimate partner violence and subsequent relationship breakdowns, and family bereavement all contributed to poor mental health and subsequent entry into sex work. In addition, the consequences of sex work such as sexual risks, and ongoing violence from police and clients, further exacerbated poor mental health.

**Conclusions:**

There is a need for both micro- and macro interventions to address poverty and violence against FSWs in Kenya, thereby reducing mental health problems. Addressing violence against women and girls may also reduce entry into sex work. Improving mental health literacy and providing mental health intervention services for ‘at-risk’ populations such as FSWs should enhance coping strategies and help-seeking efficacy.

## Introduction

According to the World Health Organization (WHO), mental health is defined as “a state of mental well-being that enables people to cope with the stresses of life, realize their abilities, learn well and work well, and contribute to their community” [1]. Poor mental health can lead to mental health conditions such as depression, anxiety and post-traumatic stress disorder (PTSD) which affect our thinking, behaviours and interaction with others [2]. The burden of poor mental health is increasing globally, particularly among key vulnerable populations such as female sex workers (FSWs). Global estimates in 2010 showed that mental ill-health, neurological disorders and harmful substance use were the leading causes of years lived with disability, accounting for 1 in every 10 lost years of health [3]. Estimates were even higher in 2016 in which 19 and 7% of years lived with disability and disability-adjusted life years were attributed to mental and addictive disorders respectively [4]. Mental health greatly affects quality of life and if untreated can lead to premature death due to ill-health or suicide [5]. The WHO estimates that there are over 700,000 suicides every year, with about 77% occurring in low-and middle-income countries (LMICs) [6]. The risk and outcome of mental health problems including suicide have been linked with a wide range of demographic, socioeconomic, neighbourhood, and relationship factors [5, 7].

FSWs are defined as girls and women who regularly or occasionally receive money in exchange for sexual services [8]. Entry into sex work may be precipitated by negative psychological events such as separation from parents, intimate partner violence (IPV), and childhood violence [9–11]. Sex work itself increases vulnerability to structural factors such as marginalization, stigma and discrimination, and gender inequality [12]. Sex work also involves occupational hazards such as unlawful police arrest, violence, deception, human immunodeficiency virus (HIV), other sexually transmitted infections (STIs), and harmful alcohol and other substance use [12, 13]. Both structural and occupational risks can predispose FSWs to psychological health problems and suicide [5, 12, 14–16]. A recent review and meta-analysis of mental health among 18,524 FSWs in LMICs reported the pooled prevalence of various mental health problems, with high estimated levels of depression (41.8%), anxiety (21.0%), PTSD (19.7%), physiological distress (40.8%), mood disorders (28.8%), recent suicide ideation (22.8%) and attempts (6.3%) [5].

The WHO estimated in 2011 that 6.6% (urban) and 5.1% (rural) of women in Kenya had exchanged sex for money in the past year [17]. Indeed, findings from the baseline quantitative Maisha Fiti study in Nairobi found that one quarter (25%) had either moderate or severe depression or anxiety and 14% had PTSD [18]. These common mental health problems were associated with a range of factors including poverty, violence and increased harmful alcohol/drug use [18]. Violence against FSWs is prevalent in Kenya with a recent survey among 220 FSWs in Nairobi reporting that 81 and 79% of respondents had experienced client-perpetrated and intimate partner perpetrated violence, respectively, in the past 12 months [19]. This study reported a high prevalence of depression (56.8%) and generalised anxiety (39.1%), both of which were independently significantly associated with a recent violent experience [19]. FSWs in Kenya are also at high risk of violence from the police with respondents in a survey in 2015 reporting having experienced high levels of arrest for selling sex (62%) as well as verbal (59%) and physical (45%) abuse at least once in the past 12 months [20]. Violence against FSWs is associated with higher levels of psychological stress and has been linked with negative health effects such as increased HIV [21, 22]. In addition, alcohol use among FSWs in Kenya is common, with about 66 and 34% of FSWs engaging in hazardous and harmful drinking, respectively [23]. Alcohol consumption among FSWs has been linked with higher risk sexual behaviour, poor uptake of HIV services, and increased risk of violence and mental health problems [5, 23, 24].

Despite the substantial body of evidence demonstrating the increased risk of violence, police arrest, and harmful alcohol use among FSWs in Kenya, there is a paucity of research on their mental health experiences and needs. A limited number of quantitative studies have investigated mental health and associated risk factors among FSWs in Kenya [19, 25, 26], including quantitative research from the baseline Maisha Fiti study, but there is little research using qualitative research methods to understand FSWs’ mental health experiences and determinants. This paper uses qualitative in-depth interviews with FSWs enrolled in the Maisha Fiti study in Kenya to investigate their lifetime mental health experiences and perceived risk factors. Findings from this study will add to the existing literature by providing contextual and in-depth interpretations of the mental health findings obtained in epidemiological studies.

## Methods

### Study setting

This study was conducted in Nairobi, Kenya’s capital city. The population in Nairobi in 2019 was estimated to be 4.4 million people, and about half were female [27]. Although poverty in Kenya is high (36%), there is a huge poverty gap between counties [28]. For example, the population living below the poverty line ranges from 16.7% in Nairobi to 68% in counties in the North Eastern province [28]. Due to economic reasons, migration from rural areas to Nairobi in search of work is a common phenomenon, especially among youth. Despite the city of Nairobi recording the lowest poverty rate, poverty and living conditions in informal settlements are far worse than in the rest of the city, with women disproportionally affected [28]. Nairobi also accounts for the highest number of FSWs (about 40,000) in the country, with approximately 2000 ‘hotspots’ where women sell sex [29]. There are different types of hotspots including bars (most common), guest houses, particular streets, and uninhabited buildings. Compared to other counties, FSWs in Nairobi have a strong network of self-help groups and support from the Sex work Outreach Program (SWOP) clinics, which provide health services and psychological support free of charge.

### The Maisha Fiti study

The Maisha Fiti study is a longitudinal mixed-methods observational study enrolling FSWs in Nairobi County, Kenya. A key objective was to assess the prevalence, severity, and frequency of FSWs’ experience of violence, mental health morbidity, and problematic alcohol and other substance use, and how these differ by HIV status. The data collection was in 3 phases, baseline (Jan-Dec 2019), midline (Jan- March 2020), and endline (June 2020-Jan 2021). However, qualitative data were collected at baseline and endline only. This paper uses baseline qualitative data to explore FSWs’ lifetime mental health experiences.

### Study site, data collection, and management

Since 2005, Partners for Health and Development in Africa have been managing SWOP clinics providing services to FSWs in Nairobi through funding from the Centre for Disease Control-President’s Emergency Plan for AIDS Relief. The clinical services provided by SWOP include HIV, STI, and tuberculosis testing and treatment, as well as the provision of condoms, counselling, and drug and alcohol risk management services. At the time of the Maisha Fiti study, there were seven SWOP clinics in Nairobi County, each of which had at least two clinical staff, a pharmacist, two HIV testing and treatment counsellors, a team of outreach workers and peer educators, and two peer advocacy violence workers. About 33,000 FSWs have enrolled in SWOP (biometric registration with fingerprints), 16,000 of whom were in active follow-up at the time of this study. Sample size calculations and participant selection have been described in detail elsewhere [18]. In brief, 1200 participants were randomly selected from a list of all FSWs (though the SWOP clinic attendance lists of clinic barcodes) who had attended any of the seven SWOP clinics within 12 months preceding the start of the study, were 18–45 years old, not pregnant or breastfeeding, and had no chronic illness such as diabetes, rheumatoid arthritis, asthma, or TB infection in the past 6 months. Information about the Maisha Fiti study was disseminated to SWOP clinic attendees through study peer educators and community mobilizers based at the SWOP clinics. The study staff initially contacted selected FSWs by telephone and explained the study. Interested women were invited to attend the study clinic in downtown Nairobi where they were provided with detailed study information (both written and verbally). Those who were eligible and consented were enrolled in the behavioural-biological survey and were also advised that they may be additionally invited to participate in in-depth qualitative interviews at a future date.

Prior to the study start, all study team members (including peer educators) participated in a three-week training programme which included modules on violence and mental health, and how to respond to distress during an interview. A referral pathway was developed for participants reporting recent violence, mental health problems or suicidal behaviours during the quantitative or qualitative survey interviews. This included an immediate referral to a specialist counsellor employed in the clinic as part of the study team, as well as onward referral to specialist psychiatric and violence intervention services on an as-needed basis. The study team also provided depression (Healthy Activity Programme) [30] and harmful drinking (Counselling for Alcohol Problems) [31] brief psychological intervention training to HIV testing and treatment counsellors based at each of the seven SWOP clinics and participants could access additional support from these counsellors if they chose to.

Of the 1003 FSWs randomly selected for the behavioural-biological survey, 40 were randomly selected and invited to participate in the qualitative in-depth interviews. The in-depth interviews followed a semi-structured interview guide and were carried out by two trained Kenyan female researchers fluent in both Kiswahili and English. Participants were asked to detail their life stories, including narrating specific life events such as entry into sex work, experiences of violence, mental health experiences, use of alcohol and other substances, as well as the use of pre-exposure prophylaxis, post-exposure prophylaxis and antiretroviral. To avoid language barriers while responding to questions, participants were asked to choose the language they were most comfortable with (Kiswahili and/or English). This helped minimize the risk of losing participants’ intended meaning or misinterpretation, therefore increasing the validity of the data collected. Despite most participants choosing to mainly communicate in Kiswahili, all the interviews were a mixture of Kiswahili and English.

All recordings were uploaded to a secure server at Partners for Health and Development for Africa (PHDA) and then destroyed. Interview transcripts did not include participant names. All interview documents were stored on secure servers at PHDA and the London School of Hygiene and Tropical Medicine. These were password protected and access was restricted to qualitative research team members only.

### Conceptual framework

**Fig. 1:**
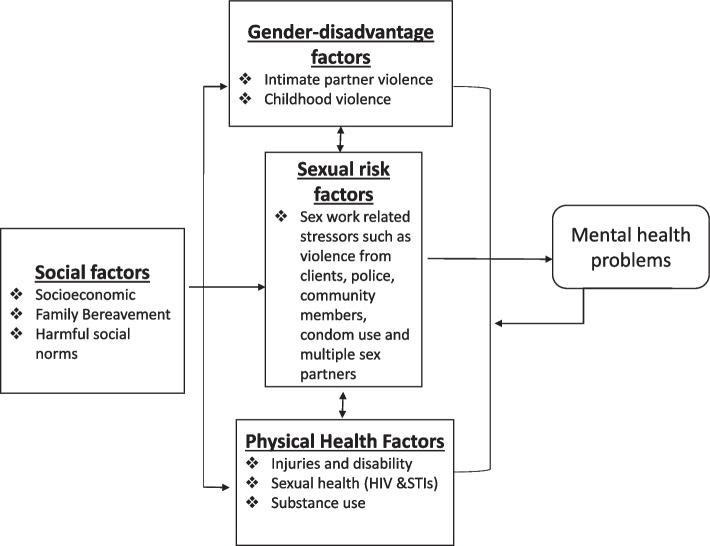
A conceptual framework for the social risk factors for subsequent mental health problems among FSWs in Nairobi, Kenya adapted from Shahmanesh et al.

When exploring suicidal behaviours among female sex workers in Goa, Shahmanesh et al. [32] proposed that social factors, gender-disadvantage factors, sex work related sexual risk factors and physical health factors were distal determinants of poor mental health and suicide. For this study, we adapted this hierarchical conceptual framework based on a review of the literature and from our quantitative mental health findings which drew on eco-social and life course theory [18, 33]. We hypothesised that socio-economic factors, familial factors, harmful social norms, gender-based violence, sex work related violence and risk, physical and sexual health factors and substance use, were all potential determinants affecting FSWs’ lifetime mental health experiences (Fig. 1). We used the framework to qualitatively explore FSWs perceived risk factors of poor mental health, both before and after their entry into sex work. The conceptual framework informed the analysis of the data and the presentation of the results.

### Data analysis

The analysis in this paper focused on participants’ understanding of mental health through their definitions of mental health and depression and their experiences and perceived risk factors of poor mental health. The interviews were recorded, transcribed verbatim, and translated into English. Data were analysed thematically, and this started while interviews were still ongoing. During data collection, daily debrief meetings with the qualitative interviewers, the study PIs, the study coordinator, and the study research assistants, provided a multi-disciplinary, multi-cultural, and multi-linguistic platform to explore women’s experiences of sex work and mental health in the Kenyan political, socio-economic, and socio-cultural context. These sessions were seminal in identifying themes of importance as well as topics that needed further probing in subsequent interviews. Following each interview, the interviewer wrote detailed notes about the interview, including observations which would not be captured during the audio-recording (such as a participant’s demeanour). These formed the ‘scripts’ which complimented the transcribed transcripts. Through the knowledge gained during the debrief meetings, the team started identifying emerging patterns and ideas of interest from the data. The scripts and transcripts were imported into Nvivo (V.12) for data organisation, coding and managing thematic analysis. Inductive data-driven codes were identified from the initial interviews, and then organised into themes and sub-themes guided by the conceptual framework. The coding framework was updated, and coding revised iteratively. Data interpretation was further explored in a series of collaborative analytic working groups. The translated words of participants are presented as quotes and used to illustrate key themes. The socio-demographic characteristics and HIV status of the participants displayed in Table 1 were obtained from the baseline behavioural and biological survey data of the Maisha Fiti study respectively. The behavioural survey captures data on socio-demographic, sexual behaviour, recent alcohol, and drug use, sexual and reproductive health, past and recent violence experience, mental health morbidity (depression, anxiety, suicidality, PTSD), and intravaginal washing practices, using validated tools as previously described (reference) The biological tests included HIV, STIs, pregnancy, systemic inflammation, cortisol levels, etc.

**Table 1:** Socio-demographics of Study Participants (*N* = 40)

Variables	N (%)
Age	
18–24	7 (17.5%)
25–34	19 (47.5%)
35+	14 (35.0%)
Raised in	
Rural	20 (50%)
Urban	20 (50%)
Education Level	
Some primary	2 (5%)
Completed primary	14 (35%)
Some secondary	10 (25%)
Completed secondary	11 (27.5%)
Higher education	3 (7.5%)
Marital Status	
Ever married/co-habited with a sexual partner	33 (82.5%)
Never married/co-habited with a sexual partner	7 (7.5%)
Current Marital Status	
Single	12 (30%)
Live-in partner	2 (5%)
Married	2 (5%)
Separated	19 (47.5%)
Divorced	1 (2.5%)
Widowed	4 (10%)
Number of Live children	
None	3 (7.5%)
1–2	26 (65.0%)
3–4	9 (22.5%)
**≥** 5	1 (2.5%)
Missing	1(2.5%)
Children born prior to entry into sex work	
None	4 (10%)
**≥** 1	29 (72.5%)
Maybe*	4 (10%)
Missing	3 (7.5%)
Age of entry into sex work **	
< 18	4 (10%)
18–24	20 (50%)
25–34	16 (40%)
35+	0 (0%)
HIV diagnosis	
HIV positive diagnosis	9 (22.5%)
HIV negative diagnosis	31 (77.5%)
HIV and entry into sex work***	
Diagnosed before entry into sex work	2 (22.2%)
Diagnosed after entry into sex work	7 (77.8%)

* had first child same year as starting sex work

** Entry to sex work is described as women regularly starting to have sex for money

*** Responses of the nine women who were HIV positive

### Ethical approval and consent to participate

The Maisha Fiti study was ethically approved by the Kenyatta National Hospital – University of Nairobi Ethics Review Committee (KNH ERC P778/11/2018), the Research Ethics Committees at the London School of Hygiene and Tropical Medicine (Approval number: 16229) and the University of Toronto (Approval number: 37046). Participation in the study was voluntary and women provided written informed consent before enrolling. Due to the potential societal marginalisation of FSWs and the sensitiveness of the topics discussed, maintaining confidentiality was a key part of the study team training. Anonymised study numbers were issued for each participant and pseudonyms were used in place of real names during interview transcription. Women were interviewed during their visit to the SWOP study clinic in a private space in which their conversations could not be overheard and were reassured of the confidentiality procedures at the start of each interview.

## Results

### Study population

The 40 FSWs interviewed had a mean age of 32 years (range: 21–44 years) and half were brought up in rural areas. All participants had some form of basic education, 11 participants had completed secondary school and three received higher education. Thirty-three participants had been married or had cohabited with a sexual partner. However, at the time of the interview, only four were married or were living with a partner. Furthermore, half of the study participants commenced sex work between 18 and 24 years old and four started before age 18. Most participants had children and the majority of them had their first child before entry into sex work. Nine participants were HIV positive with seven testing positive after entry into sex work (Table 1).

### Understanding of mental health

In terms of participants’ knowledge of mental health, five said they had no idea how to define mental health. For example, when one participant was asked what comes to her mind when she hears about mental health, she said:

*“I do not know. Does it mean someone is healed?”* ***(MF 113).***

However, although most participants could not provide a clear definition of mental health, based on their personal and second-hand experiences they could link it with stress (Dhiki, *n* = 23), depression (Msongo wa mawazo, *n* = 17), insanity (Pagawa, *n* = 16), suicide (Kujitia kitanzi, *n* = 6) and harmful substance use (Madawa za kulevya, *n* = 3). Other descriptions included someone being mentally disturbed or having mental issues (kusumbuka akili) and excessively ruminating (kufikiria sana). Respondents who were interviewed either wholly or partially in English also defined mental health as ‘craziness’ or ‘madness’ (referring to ‘insanity’), described as being hospitalised or wandering the streets collecting rubbish. However, other participants linked it with witchcraft (Uchawi/urogin = 7) or physical illnesses (Ugonjwa wa kimwilin = 2) such as STIs (Magongwa ya zinaa) and diabetes (Kisukari). The words in brackets were the local terms they used to describe mental health.

The quotes below illustrate how study participants defined mental health, with some linking it to mental health problems such as stress, insanity, and suicide:

*“[it is]a person who is insane. I am okay because I am not crazy. People are known to be crazy when they start walking naked or speaking to themselves” (MF 186).*

*“Maybe you have piled too much stress in your mind that you are defeated you don’t have direction, you are just there. You could even be crossing, and you get hit by a car and you are just there. It is like you are there and you are not there” (MF 497).*

*“They become mad. Let us say someone has disagreed with her husband. Her husband chased her with all the children, and you do not know where to begin, food etc. and you do not have a job, thoughts come to your mind…until you think of committing suicide”* ***(MF 208).***

Participants were also specifically asked if they could define ‘depression’ and most linked it with stress, with several respondents using the two terms interchangeably and often described as ‘thinking too much’ (Kufikiri sana). However, some participants (*n* = 6) distinguished between mental health as a clinical condition and depression as societal stress. Below are the responses from two participants who were asked if depression is a mental illness:

*“Depression is when one is stressed. While mental sickness is when one is insane. With mental sickness, the brain is not functioning well. While with depression the brain is functioning okay, but you are stressed”* ***(MF 113).***

*“It is not madness. It is something disturbing her in the mind but not madness. There is a problem”* ***(MF 017).***

### Determinants of mental ill-health

Of the 40 FSWs interviewed, 28 described personal experiences of poor mental health, whether described as mental health issues, stress, depression, or PTSD; seven women reported previous suicidal ideation. All but one of these participants narrated the factors that they perceived precipitated the mental health episode. The most reported risk factor was intimate partner violence, followed by poverty, sex work-related risks such as violence from clients, or the death of a family member. Infrequently mentioned factors included physical disability and harmful substance use. We mapped the reported risk factors against i) social factors, ii) gender disadvantage factors, iii) sexual risk factors and iv) physical health, as the four main distal determinants of suicide and mental health among FSWs, depicted by the conceptual framework Fig. 1.

I) Social Factors

The main themes that emerged in relation to social risk factors for poor mental health were poverty and family bereavement. These findings are presented below.

### Poverty

Poverty among FSWs was described as a key social determinant influencing poor mental health and thoughts of suicide among FSWs. Out of the 28 respondents who narrated their own mental health experiences, eight of them specifically linked it with poverty. Almost all of them were once married or cohabited with a partner and had one or more children, although none of them received child support from the fathers of their children after being divorced or separated. Relationship breakdown plunged them into poverty. Several of them had no parents and received no support from relatives. As the sole source of income, respondents struggled to provide basic needs for themselves and their children, such as food and school fees, and they struggled to care for other family members. Some respondents reported having to borrow money to provide for their children’s basic needs:

*“Those are very tough times because issues follow each other like you have not paid rent, books are needed and no food. It can really be very bad. I am alone without a penny and I have nobody to assist me, so I look for where to borrow money”* ***(MF 033).***

Reports of mental health problems such as suicidal thoughts, being stressed and depressed due to financial challenges were frequently narrated:

*“I had thought I will buy poison; I kill all my children and I kill myself and life would end. Now I did not have even the money (to buy poison). Now I said if I get even thirty bob (shillings), I will ask how much Rat kill is, and I give all my children and I drink also and we all die at night”* ***(MF 497).***

*“Sometimes I feel like running away from that house. Because you find that there is nobody to help me. Then I ask myself, if I go my grandmother will struggle so much and she might die. Here again is my younger brother, what will happen to him if I go? There are times you get very broke like weekdays. You wonder what to do because, these old people are looking up to you; they need to eat, to pay rent, yes”* ***(MF 240).***

Furthermore, as highlighted by the conceptual framework, poverty influences other determinants of poor mental health such as higher risk sexual behaviours and increased risk of violence (Fig. 1). Findings in this study demonstrated that most of the women who reported poverty as the main cause of their poor mental health experiences explained how financial stress and their desire to care for their children, motivated them to start sex work. Several of them did not complete their secondary education, could not find a better job for survival, and received no financial support. Their only option was to sell sex for ‘quick money’, which had the potential to further increase their vulnerability to poor mental health:

*“You see I was not getting support from anybody and remember my mother had thrown me out maybe if she supported me, I could not have gone into sex work. By the way, this job I was not introduced to it by anybody. I was not very bright, and this was the only way out to get quick money. So, I started going to the bar and that is how I started sex work”* ***(MF 033).***

### Death of a family member

The impact of the death of loved ones on FSWs’ mental well-being surfaced in the interviews. Some respondents reported symptoms of stress and depression following the death of a family member especially when the deceased had provided financial support. Reports of financial stress after the death of a spouse or parent(s) were recounted by some women, this precipitated their entry into sex work as they had no other means of survival. For example, when one of the participants was asked about depression, she explained:

*“Me I would say I am like that (that she is depressed). I used to have so many more thoughts than normal because I don’t have my people. Here is my child and I had to do this job (sex work). Sometimes when I think about it, I just cry. I would imagine if I had my parents or my family, I would not do this work”* ***(MF 0208)****.*

Another participant wept during the interview as she told how she was alone with her children when her husband who used to provide for them had died:

*“By 2017 my husband died and left me alone: I am alone bringing up my children [weeps]. It has been very difficult because when my husband died, they (husband’s relatives) took everything from me and only left me with a bed”* ***(MF 0547).***

As a coping mechanism, a participant explained that she had started using cannabis to assist her to recover from the tragic death of her husband who was murdered by an unknown assailant:

*“Then I would cry a lot, nowadays I don’t. Plus I also learnt something, you know when you go through so much pain you get something to relieve yourself. I smoke a lot of cannabis, me I smoke a lot of cannabis. It helps me with those challenges now”* ***(MF 0569).***

The family of her late husband accused her of being the killer of her husband, which added to her stress.

II) Gendered Disadvantage

Intimate partner violence was the most reported gendered disadvantage causing mental health problems among respondents.

### Intimate partner violence (IPV)

All the IPV-related mental health problems narrated by respondents occurred during their previous relationships before sex work commencement. Almost half (*n* = 11) of the participants who reported experiencing poor mental health linked it to their previous experiences of IPV. Previous IPV also accounted for the highest number of participants reporting suicidal thoughts (4 out of 7). Other mental health symptoms due to IPV were also reported, such as depression, PTSD and living in fear.

Although respondents narrated the experience of IPV in all forms (i.e., physical, verbal, emotional, economic, and sexual violence), physical violence was the most frequently reported. Several women described how they were beaten and verbally abused by their previous intimate partners. For example, one woman explained how she was beaten by her husband until he broke her leg. Respondents described social norms that sanction a man’s right to assault a wife physically and sexually, and that society expects women to endure the pain husbands inflict. As such, some described how they had to stay in their marital homes with abusive partners until they could not handle the stress of the marriage anymore. All the 11 respondents who reported IPV from their previous partners either asked for a divorce or escaped from their marriages/relationships to start a new life with their children.

The quotes below illustrate respondents’ explanations of how their IPV experiences affected them mentally and led to suicidal thoughts:

*“Instead of giving you love, he gives you beating. So when you reflect you even have suicidal thoughts like I used to look at myself and wonder I don’t have a mother and I am suffering with these children I feel that death was a better option” (****MF 0393).***

*“I would feel like even killing myself. I used to think about going back home but I told you that my grandmother used to tell me that a woman is to endure. Now I am enduring, and if I go home what will I say”* ***(MF 520).***

Reports of being frustrated and stressed due to IPV were also described:

*“I even looked old because of stress and many frustrations. This person (husband) is with you in the house and he does not want to look for a job other than sitting at home. So you even wonder what is happening. Just try asking whether he will go to work and he will rain blows on you”* ***(MF 0004).***

Furthermore, because of the notion that a woman should endure pain, and since several respondents were either orphans or raised by a single parent, they could not rely on family members so had to look for their accommodation and survive alone after separating from their husbands. All women except one had children with their intimate partners so starting life over again with their children was a challenge as they received no support from the fathers of their children. This resulted in several of them entering sex work for survival since most had not completed school and couldn’t find a job that could provide for their needs and that of their children. Despite their financial struggles, several respondents vowed never to return to their ex-husbands:

*“We (respondent and children) used to sleep down on the mattress because I did not have a bed and I had sworn that come rain or sunshine I can never go back to that man who had married me. I needed peace and so I could not go back. That is how my job started (sex work) and I would go to the clubs looking for clients and I did very well and I was able to support my child”* ***(MF033).***

III) Sexual risk factors

In terms of sexual risk factors, issues related to sex work were the most commonly reported theme and it was the third most reported perceived cause of poor mental health experiences among respondents. Almost all respondents who related their poor mental health to sex work-related risk factors either got into sex work due to poverty, mainly after the experience of IPV or the death of close family members. This illustrates the interrelationships between the distal factors of suicide and mental health as illustrated in the conceptual framework.

### Sex work-related

Despite sex work being a good source of income for most respondents, it was noted as a stressful and risky job. Some respondents described sex work-related mental health challenges, such as violence from clients refusing to pay after sex or being beaten and forced into condom-less sex. In addition to risks from clients, reports of the city Askaris (police) chasing FSWs and demanding to be bribed in cash or through sex were narrated. These experiences significantly affected respondents psychologically:

*“When you are arrested by the Council (police) it is a risk because you will be arrested and the Council police will want to sleep with you, he sleeps with you and he does not pay you and he will still take you to court”* ***(MF 0012).***

*“Once you are found on the streets walking, whether it is the police or Council you will have to go (being chased away)”* ***(MF 0012).***

One respondent narrated being stressed and living in fear for her life due to her work:

*“Me I tell you this sex work job, sometimes it gives me stress. If I had been stabbed or hurt, I think about if I die because of this job who would be left with this child. I just think about many things”* ***(MF 0208).***

In addition, the mental health impact of not knowing one’s HIV status after forced condomless sex with a client was narrated by two respondents. One of the respondents said she did not go to the hospital when the incident happened as she was not aware of her options to reduce her risk of HIV. However, the other respondent did go to the hospital for post-exposure prophylaxis although she struggled to take the pills as she found them too big. She threw them in the toilet when her client tested HIV negative. Below are the quotes for the two respondents narrating their mental health experiences following a condomless sex encounter:

*“Basically I was unhappy and restless. I was stressed so much so people noticed. He (regular client) even noticed and would tell me ‘babe you seem so stressed’, I told him it’s because I didn’t know my status or yours”* ***(MF 0113).***

*“I felt like my heart was burning. It affected me so much because I was wondering whether he had infected me with the virus, or with sexually transmitted diseases, or what does he want with me, is he a devil worshipper or what, uh! For a number of days, I was feeling I don’t want to go to work, I would stay like that bored”* ***(017).***

Lastly, sex work also exposes FSWs to violence and stigma in their communities, which is the reason why a lot of FSWs tend to hide their job from neighbours and family members. Reports of FSWs being physiologically affected by the stigma they experienced in their communities were narrated. The quote below shows a description of a respondent’s experience of verbal abuse from a neighbour and how that affected her:

*“Psychological (violence) is when you find someone is insulting you before your own children calling you a prostitute. ‘You prostitute’ and such before your children. So sometimes you just suffer alone”* ***(MF 0058).***

IV) Physical Health Factors

In relation to the fourth domain of the conceptual framework, only a few women related their poor mental health to physical disabilities or harmful substance use. These findings are presented below.

### Physical health and disability

A few study participants (*n* = 3) perceived poor physical health or disability as the cause of their mental health issues. This included one participant losing an eye when a client hit her with a soda bottle and another who had an elongated growth around her labia, which she linked with reconstructive surgery she had after being raped at 3 years old. Interestingly, the stress of living with HIV was mentioned by only one participant.

The woman who lost her eye described how her disability affected her mental wellbeing:

*“I was used to using my two eyes so after I lost one eye it was very stressful when crossing the road because I was not used to using one eye to check if the road is clear. I used to get very stressed and like where I stayed, there was a highway so I used to wait for people so that we can cross the road together and other times I could hold someone’s hand so that we cross the road together. It took me a while to get used to using one eye and I was stressed for about one year to acclimatize” (MF033).*

#### Harmful substance use

Although several women described their use of alcohol and other harmful substances such as cannabis and bhang for courage and as a coping mechanism while at sex work, only one participant explicitly linked it to poor mental health. The participant claimed to have nearly killed her child due to drug use:

“*I held that child and I wanted to kill him, I held a knife and said this thing why is it stressing me. When I held the knife like this, I felt a sharp cut in my heart, my senses came back and I asked myself, God, what do I want to do? So I was like if I kill this child, I am the one with a problem”* **MF 423).**

The woman panicked and she promised herself to never smoke cannabis again.

## Discussion

Our findings demonstrate that poor mental health is both a driving factor on the pathway into sex work and a consequence of sex work for many women. We show that factors such as poverty, low levels of education, poor job opportunities, family bereavement, the lack of family support, maternal responsibilities, harmful gender norms, IPV, and subsequent relationship breakdowns, all contribute to poor mental health and subsequent entry into sex work in Kenya. Although sex work helps women mitigate some of the factors that influenced their entry into the industry, such as providing an income and enabling women to support their families, the sexual risks involved in sex work, ongoing violence from police and clients, and, in a few cases, the physical harm and harmful substance use consequences of sex work, exacerbate poor mental health.

In this study, most participants defined mental health in terms of psychotic health conditions such as insanity, to the exclusion of psychological health conditions such as stress. However, although few women specifically defined symptoms as mental health conditions, some participants could describe symptoms of stress and depression. These findings are consistent with previous studies in Kenya with youth and the general community, which noted that members may not know the terms for specific mental health problems but can describe them based on symptoms [34, 35]. Our study also highlighted the belief in supernatural causes of mental ill-health, which has been demonstrated previously with the general population and is linked to patients and their carers seeking pluralistic treatment from traditional and faith healers, as well as biomedical healthcare providers [35, 36]. Poor mental health literacy is known to be one of the major barriers to seeking professional biomedical mental health care in the sub-Saharan Africa (SSA) [37]. For example, community-based mental health literacy radio programmes in Malawi and Tanzania showed significant improvements in knowledge, and mental health-seeking behaviour among youths in the intervention targeted areas compared to those who were not exposed to the intervention [37].

Our study demonstrates that FSWs in the Maisha Fiti cohort are vulnerable to several mental health challenges. This is consistent with the high burden of poor mental health that was demonstrated quantitatively in the larger study cohort [18]. A substantial proportion of participants had experienced mental ill-health before entering sex work. Poor mental health as a driver on the pathway into sex work was linked to stressful life events such as marital breakdowns related to IPV, lack of family support, maternal responsibilities, poverty, and lack of other opportunities. IPV in Kenya is closely linked with gender inequality and harmful social norms, compromising women’s ability to have autonomy over their bodies and to resist, or in many cases especially in marriage, seek protection from, or recourse in response to IPV [38]. The psychological distress of experiencing IPV was a key factor in participants seeking a divorce. However, divorce in the absence of financial support or viable employment options, particularly in the context of children to feed, was frequently a driving factor that pushed women towards sex work. Interestingly, all the IPV-related mental health problems narrated by respondents in this study occurred before sex work commencement. A possible reason for this could be because most of the women interviewed had divorced or separated from their abusive most-significant relationships to date before becoming sex workers. It may also be due to social desirability bias, and the women wishing to portray themselves as having escaped from such difficult relationships.

Poverty is a well-established risk factor for poor mental health [39, 40] both directly and indirectly, by increasing the risks of food insecurity, IPV, and entry into sex work [5]. The distress of food insecurity and women’s desire to provide for their children’s basic needs consistently emerged as a driver into sex work and has been reported elsewhere [41–43]. Loss of financial support due to the death of a close family member such as a parent, a partner or after a divorce is a stressful event that not only mentally affects women but also a push factor into sex work commencement to cover household needs [44]. Poverty is the main driver pushing women into sex work across the globe, particularly in SSA [41, 45, 46].

In Kenya, the legal status of sex work is complex. It is not criminalised by federal law but may be prohibited by municipal by-law, as is the situation in Nairobi county [47, 48]. This quasi-criminalised nature of sex work, combined with structural factors such as social stigma and poor working conditions, increases the risk of violence against FSWs and deters them from seeking justice if/when violence occurs [49, 50]. Violence from clients and police is common for FSWs. Client-related violence most frequently occurs concerning negotiations around condoms and payment [49]. Whereas participants’ experience of violence or coercion from the police mostly occurs in the course of the police enforcing sex work as unlawful. Thus, police exploit FSWs due to the unlawful status of sex work, by demanding bribes, sex, and confiscating items such as money from FSWs who often fear being arrested due to lack of legal protections. Reports of FSWs being unlawfully arrested, tortured, sexually abused, and assaulted by the police have been documented in this study and others conducted in Kenya [51]. Police exploitation has been associated with an increased risk of violence from clients, for example, forcing FSWs to hurriedly accept clients without properly negotiating terms and thereby increasing the risk of fractious agreements and the potential for violence [52]. In addition, aggressive policing tactics have been shown to force street-based FSWs to relocate to unfamiliar and less safe places where they are at higher risk of violence, such as being forced into sex without condoms by clients, and robbery or rape by criminals [53, 54]. These not only mentally drain FSWs but also increase their risks of HIV and other STIs.

Physical health factors occurred due to the impact of the higher-risk sex work environment and were not reported as drivers into sex work. For example, physical disability resulting from the experience of physical violence from a client has been reported in this study. Physical disability affects engagement in productive activities, leading to poor mental health and quality of life [55]. Moreover, HIV infection is also known to predispose to increased physiological and psychological health problems among FSWs [5]. Although a known association, it was only mentioned by one participant in this study. This is similar to the findings among the Maisha Fiti study cohort showing no evidence of an association between HIV and mental health problems [18]. Possible explanations for the low mental health impact of HIV among HIV-infected respondents could be that they were less affected by their HIV diagnosis due to access to free Antiretrovirals (ARVs), or due to other social support provided by FSW Community-Based Organisations (CBOs) and SWOP peer educators, which helped them cope with their HIV status [56, 57]. Interestingly, despite substance use being a well-established risk factor for poor mental health and commonly utilised by participants to cope with the challenges of sex work, only one of the respondents mentioned drug use as a perceived risk factor for their mental health problems. Findings from the larger Maisha Fiti cohort showed evidence of an association between harmful alcohol/drugs and common mental health problems although the direction of causality could not be ascertained [18]. The low report of alcohol/drug-related mental health problems could be due to social desirability bias or participants’ poor knowledge of harmful alcohol and drug use as potential risk factors for poor mental health. These may make them only narrate their mental health experiences of other stressful events such as poverty, which has been associated with both harmful alcohol/drugs and common mental health problems [18, 58]. Alternatively, most of the women interviewed may not be harmful alcohol/drug users.

Overall, the key findings in this study are the profound mental health impacts of poverty and violence on the lives of women and girls in Kenya: these need urgent attention to reduce the risks of entry into sex work and sex work-related mental health problems. Poverty alleviation strategies such as micro-finance interventions may benefit women and girls who have experienced negative events such as divorce or are single mothers, thereby reducing the risks of entry into sex work. Micro-finance interventions empower the poor, particularly women, and are known to be more effective when combined with other strategies such as better education, development training and other livelihood enhancement measures [59]. Similarly, with evidence that violence prevention and mitigation interventions are effective for women and FSWs in SSA [60–62], such interventions should be set in place in Kenya with tactics to ensure that women who experience violence are referred for mental health support. This can be addressed in parallel with community-based awareness campaigns against harmful social norms associated with gender-based violence.

This study had limitations. Due to the sensitive nature and stigma attached to mental health, respondents were not directly asked if they had experienced mental health challenges. Their mental health experiences were captured through probing how specific events in their lives affected them psychologically. Therefore, there is a possibility that some participants’ mental health experiences were not captured. Nonetheless, even if they had been asked directly some women would not have responded either because they may not have considered some events as mental health issues or may not have wished to talk about them. The interviewers in this study were highly trained in probing to answer the Maisha Fiti research aims when permitted by respondents. Furthermore, responses may have been subjected to social desirability bias to sensitive topics such as sex work and harmful substance use. However, the main strength of this study was that women were recruited from the SWOP program that had established and trusted relationships with the FSWs, therefore limiting the risk of social desirability bias. Trust is known to reduce response bias by motivating respondents to engage in more open and honest discussions [63, 64]. Another strength of this study is that participants were randomly selected from a larger cohort of FSWs in Nairobi, thereby increasing the generalisability and validity of the findings in the study. Lastly, although participants’ perceived mental health risk factors evidenced in this study fitted the four distal determinants of mental health and suicide illustrated by the hierarchical conceptual framework, findings cannot be generalised to the whole population of FSWs in Nairobi due to methodological limitations. Further research using the conceptual framework of this study to test for FSWs’ mental health experiences in larger studies is therefore recommended.

## Conclusion

Our work has shown that FSWs in Nairobi are vulnerable to mental health problems including suicidal thoughts. The current study demonstrates that poor mental health is not only a consequence of the higher risk sex work environment among FSWs but – along with IPV, relationship breakdown and poverty – is also a driving factor for entry into sex work. Therefore, this calls for both micro and macro interventions to address key structural drivers such as poverty and violence. There is also a need to focus on mental health literacy among vulnerable populations like FSWs and other women experiencing stressful life events. This may provide them with the knowledge to prevent and cope with common mental health problems as they arise, reduce stigma, and enhance mental health help-seeking efficacy.

## Data Availability

The datasets generated during and/or analysed during the current study are available from the corresponding author upon reasonable request.

## Abbreviations

FSWs: Female Sex workers
HIV: Human Immune Virus
IPV: Intimate partner violence
LMICs: Low-and middle-income countries
PTSD: Post-traumatic stress disorder
STIs: Sexually Transmitted Infections
SSA: Sub-Saharan Africa
SWOP: Sex Workers Outreach Programme
WHO: World Health Organisation
